# Host-Specific and Environment-Dependent Effects of Endophyte *Alternaria oxytropis* on Three Locoweed *Oxytropis* Species in China

**DOI:** 10.3390/jof11070516

**Published:** 2025-07-09

**Authors:** Yue-Yang Zhang, Yan-Zhong Li, Zun-Ji Shi

**Affiliations:** State Key Laboratory of Herbage Improvement and Grassland Agro-Ecosystems, College of Pastoral Agriculture Science and Technology, Center for Grassland Microbiome, Engineering Research Center of Grassland Industry, Ministry of Education, Lanzhou University, Lanzhou 730020, China; zhangyueyang21@lzu.edu.cn

**Keywords:** endosymbiont, photosynthesis, swainsonine, locoweed, abiotic stress

## Abstract

Plant–endophyte symbioses are widespread in grasslands. While symbiotic interactions often provide hosts with major fitness enhancements, the role of the endophyte *Alternaria oxytropis*, which produces swainsonine in locoweeds (*Oxytropis* and *Astragalus* spp.), remains enigmatic. We compared endophyte-infected (E+) and endophyte-free (E−) plants of three main Chinese locoweed species (*O. kansuensis*, *O. glabra*, and *O. ochrocephala*) under controlled conditions, and analyzed environmental factors at locoweed poisoning hotspots for herbivores. The results demonstrated significant species-specific effects: E+ plants of *O. glabra* and *O. ochrocephala* exhibited 26–39% reductions in biomass, net photosynthetic rate, and stomatal conductance, with elevated CO_2_ levels, while *O. kansuensis* showed no measurable impacts. Swainsonine concentrations were 16–20 times higher in E+ plants (122.6–151.7 mg/kg) than in E− plants. Geospatial analysis revealed that poisoning hotspots for herbivores consistently occurred in regions with extreme winter conditions (minimum temperatures ≤ −17 °C and precipitation ≤ 1 mm during the driest month), suggesting context-dependent benefits under abiotic stress. These findings suggest that the ecological role of *A. oxytropis* may vary depending on both host species and environmental context, highlighting a trade-off between growth costs and potential stress tolerance conferred by *A. oxytropis*. The study underscores the need for field validation to elucidate the adaptive mechanisms maintaining this symbiosis in harsh environments.

## 1. Introduction

Endophytic fungi deeply influence plant physiology, development, and metabolism, which has become an indispensable subject in plant research [1]. Some endosymbionts, such as *Epichloë* spp. in Poaceae, exhibit clear mutualistic benefits like enhanced drought tolerance and herbivore resistance [2,3,4,5], while numerous studies have revealed that endophyte impacts can be neutral or even detrimental [6]. For example, ryegrass (*Lolium perenne*) infected by *Neotyphodium lolii* exhibited significantly lower biomass, reduced water use efficiency, and greater wilting compared to endophyte-free plants [7]. Similarly, *N. occultans* in *L. multiflorum* acts as either a beneficial or harmful symbiont depending on environmental stress: it is detrimental under low-stress conditions and beneficial under high-stress conditions [8]. Such variability underscores the need to contextualize the effects of endophytes across host species and environmental gradients.

In locoweeds (*Oxytropis* and *Astragalus* spp.), the ecological role of *Alternaria* section *Undifilum* endophytes remains particularly enigmatic [9,10]. While *Astragalus* and *Oxytropis* species harbor diverse endophytic communities (e.g., *Chaetomium* sp.), *Alternaria* section *Undifilum* has been identified as the predominant endophyte in locoweeds, playing a pivotal role in their ecological adaptation [9,11,12]. These toxic legumes, containing the neurotoxic alkaloid swainsonine produced by their fungal endophytes, infest approximately 110 million hectares of rangelands in Western China alone, with *Oxytropis kansuensis*, *O. glabra*, and *O. ochrocephala* being particularly problematic species due to their association with *A. oxytropis* [11,13,14,15,16,17,18]. Unlike typical defensive mutualisms, in which toxins deter herbivory [19], swainsonine appears to promote sustained grazing by livestock, with poisoning symptoms manifesting only after weeks of consumption [20,21,22]. In addition, swainsonine also enhanced herbivory by specialist seed beetles (*Acanthoscelides* spp.) [23]. The beneficial role of locoweed endophytes, such as regulating the assemblage of pathogens on locoweeds, was first reported by Lu et al. [24]. *A. oxytropis* promoted lateral root and root hair development, through upregulating endogenous auxin levels and modulating the expression of auxin biosynthesis, signaling, and transport genes [25]. While *Alternaria* section *Undifilum* are vertically transmitted through specialized seed coat colonization [26], their fitness consequences for hosts remain unclear. Regarding locoweed growth, the sole available study reported that endophyte reduced the biomass of American locoweeds (*A. mollissimus* and *O. sericea*) under well-watered conditions but had neutral effects during drought [27], suggesting context-dependent outcomes. However, significant biogeographic differences exist between Chinese (endophyte *A. oxytropis* in *O. kansuensis*, *O. glabra*, and *O. ochrocephala*) and North American systems (endophyte *A. cinereum* and *A. fulvum* in *O. sericea* and *O. lambertii*) [28], necessitating region-specific investigations.

This study addresses three critical knowledge gaps: (1) whether the growth-inhibiting effects observed in American locoweeds extend to dominant Chinese species, (2) how endophyte infection influences physiological performance across different locoweed hosts, and (3) what environmental factors maintain this seemingly maladaptive symbiosis in poisoning hotspots of herbivores. Through controlled experiments comparing endophyte-infected (E+) and endophyte-free (E−) plants of three key species (*O. kansuensis*, *O. glabra* and *O. ochrocephala*), we explored impacts on host photosynthesis and biomass while analyzing environmental factors associated with the presence of beneficial symbionts. Our findings revealed a complex interaction spectrum of endophytes, demonstrating that their effects on hosts vary with both host species and environmental contexts, thereby holding significant implications for rangeland management.

## 2. Materials and Methods

### 2.1. Seed Source

The seed materials of *O. kansuensis*, *O. glabra*, and *O. ochrocephala* were collected in Inner Mongolia autonomous region, Ningxia province, and Gansu province in China, respectively.

### 2.2. Seed Coat Removed or Not

Previous studies have shown that locoweed endophyte *Alternaria* section *Undifilum* mainly exists in the seed coat of the locoweed [11]. Therefore, non-endophyte plants can be obtained by removing the seed coat. Firstly, the plump and healthy seeds of the three locoweed species (disease-free, wormhole-free, no obvious scars on the surface) were selected and surface sterilized [29]. Afterward, a scalpel was used to carefully scratch the seed coat (without damaging the seed embryo) and then soaked in sterile water for 12 h to absorb water. The seeds of each species were divided into two groups: n = 100 seeds without coats (SC−) and n = 100 seeds with coats (SC+). The seed coats of SC− seeds were carefully peeled off. The embryo of SC− seeds and whole SC+ seeds were separated and placed on the water agar medium (WA) and were cultured in a 25 °C incubator for 1 week (Figure S1).

### 2.3. Selection of E+ and E− Seedlings and Transplanting in Greenhouse

A SZX7 stereoscopic microscope (Olympus, Tokyo, Japan) was used to assess the growth of endophytic fungi on the peeled and unpeeled seeds after 48 h of culturing, and the carriage rate of endophytic fungi was calculated. The germination rates of the seeds were also calculated after culturing for 48 h. The criteria for determining *Alternaria* section *Undifilum* were as follows: the mycelium grew slowly, grew from the break of the seed coat, and was white, curved, and wavy.

$$The\;endophytic\;fungus\;carriage\;(\%)=\frac{The\;number\;of\;seeds\;with\;endophtes}{The\;number\;of\;all\;seeds}\times 100\%$$

$$The\;germination\;rate\;(\%)=\frac{The\;number\;of\;germinated\;seeds}{The\;number\;of\;all\;seeds}\times 100\%$$

### 2.4. Seedling Management and Transplanting

After 1 week of culturing the seeds on WA, the SC− seedlings without microbial growth and the SC+ seedlings with only *Alternaria* section *Undifilum* were selected (n = 45 per group). These seedlings were carefully transplanted into the prepared sterilized Danish Pindstrup peat substrate (Pindstrup Mosebrug A/S, Syddjurs, Denmark) in 250 mL paper cups using sterilized tweezers. The growth substrate had a pH of 5.8–6.2. Plants were maintained in a greenhouse under controlled conditions: 25 °C day/18 °C night temperature regime with a 12 h photoperiod provided by LED plant growth lights (dominant red light at 630–660 nm and blue light at 450–470 nm) at 150 photosynthetic photon flux density (PPFD). Additionally, the plants were sprayed with plant liquid fertilizer (Scotts Miracle-Gro Company, Marysville, OH, USA) once a week. This resulted in 6 experimental groups (3 species × 2 seed coat treatments) with 45 biological replicates each (total N = 270 plants).

### 2.5. Re-Confirming E+ and E− Plants

The plants cultivated in the greenhouse were used for isolation and PCR assays. Healthy branches of 2–3 cm were cut from the stem of four-month-old plants and longitudinally divided into two sections, which were used for isolation detection and PCR detection, respectively.

#### 2.5.1. Isolation Detection

Twelve locoweed plants growing from SC− seeds (SC− plants) were used to detect endophytic fungi. If none of the n = 12 plants had endophytic fungi, no further analysis was conducted. Otherwise, all the SC− plants should be tested, as more than one plant may contain endophytic fungi. All n = 45 plants of each locoweed species with seed coats (SC+ plants) were detected. The isolation detection was performed as follows: the stems of the locoweeds were washed under running water to remove the surface soil, and then were sterilized by the method of Liu et al. [29]. The tissues were cut into small pieces (about 3 mm), placed on potato dextrose agar medium (PDA) with sterile tweezers, and cultured in an incubator at 22 °C. The appearance of endophytic fungi from the cutting plane was observed after 24 h of cultivation. The criteria for determining endophytic fungi were as follows: the mycelium grew slowly, grew from the incision, and was white, curved, and wavy.

$$Isolation\;Detection\;(\%)=\frac{Number\;of\;plants\;isolated\;from\;endophytic\;fungi}{Number\;of\;all\;plants}\times 100\%$$

#### 2.5.2. PCR Detection

DNA of the other stem sections was extracted using the Ezup Column Plant Genomic DNA Extraction Kit (HP Plant DNA Kit D2485; Omega Bio-Tek, Norcross, GA, USA) following the operating procedures. Three centrifuge tubes with *A. oxytropis* isolated from *O. ochrocephala*, *O. glabra*, and *O. kansuensis* were used as positive controls, while a centrifuge tube without locoweed tissues was used as a negative control in DNA extraction control and PCR amplification. The locoweed endophytic fungi-specific primers OmtssuF/OmtssuR [30] were used for PCR amplification of the extracted DNA by using a PCR instrument (T100^TM^ Thermal Cycler, Bio-Rad Laboratories, Hercules, CA, USA). PCR reactions had a volume of 20 µL containing 1 μL of DNA template, 1 μL of each primer, 10 μL 2 × Es TaqMasterMix (Dye), and 7 μL of ddH_2_O. The PCR conditions were as follows: initial denaturation at 94 °C for 10 min, denaturation at 94 °C for 45 s, annealing at 52 °C for 45 s, extension at 72 °C for 45 s for 35 cycles, and further extension at 72 °C for 10 min. Agarose gel electrophoresis was used to detect PCR products of specific primers via a Bio-Rad electrophoresis instrument (PowerPoc Basic; Bio-Rad Laboratories, Hercules, CA, USA). The products were removed at 120 V, 400 mA for 30 min and imaged using a gel imaging machine (GelStudio, Analytik Jena US LLC, Upland, CA, USA) to observe whether single and clear 164 bp DNA bands were generated. The plants with a DNA band contained endophytic fungi, and those without a target band did not contain endophytic fungi.

If the locoweed plant did not carry endophytic fungi by both PCR and isolation detection, it is determined as an E− plant, otherwise it was considered as an E+ plant.

### 2.6. Effects on Plants

The survival rates, height, shoot dry weight, root dry weight, and photosynthetic parameters of the E+ and E− plants were measured after four months of cultivation in the greenhouse.

#### 2.6.1. The Survival Rates

The survival rates of the E+ and E− seedlings were recorded after transplantation to the greenhouse.

$$The\;survival\;rate(\%)=\frac{The\;number\;of\;surviving\;seedlings}{The\;number\;of\;transplanted\;seedlings}\times 100\%$$

#### 2.6.2. Height

The heights of the shoots for all plants were measured vertically from the surface of the soil using a steel ruler. The data were recorded, and the heights of three locoweed species with and without endophytic fungi were compared (n = 21 per treatment group).

#### 2.6.3. Biomass

The aerial parts of the plants were cut 2 cm above the soil surface and the underground parts of the plants were cut as roots. Both parts were dried in an oven at 80 °C until the weight no longer changed, and then the weight was determined (n = 21 per treatment group).

#### 2.6.4. Photosynthetic Parameters

The leaves of each plant were selected and labeled for net photosynthetic rate (Pn), transpiration rate (Tr), stomatal conductance (Gs), and intercellular CO_2_ concentration (Ci) analysis using photosynthetic–fluorescence system instruments (LI-6400XT, LI-COR Biosciences, Lincoln, NE, USA) from 10:00 a.m. to 11:00 a.m. (n = 10 per treatment group).

#### 2.6.5. The Swainsonine Content of E+ and E− Plants

The aerial parts of E+ and E− locoweed plants (n = 5 per treatment group) were collected in triplicate, dried indoors, ground into powder, subjected to freeze drying for approximately 24 h to remove moisture, and weighed out as 1 g. Swainsonine extraction from plants was performed using acetic acid as described by Gardner and Cook [31]. A stock solution of swainsonine standard was prepared at a concentration of 0.50 mg/mL in water, and a standard curve of swainsonine was made by gradient dilution. The samples were analyzed by an Ultra-High Performance Liquid Chromatography–Mass Spectrometry System (SCIEX, Marlborough, MA, USA) with a binary mobile phase consisting of 1% aqueous formic acid (A) and methanol (B). The gradient elution program was set as follows: 0–0.5 min, 90% A; 0.5–3 min, 90% A→5% A. The flow rate was maintained at 0.3 mL/min through a C18 column (2.1 × 50 mm, 1.7 μm) thermostated at 30 °C, with an injection volume of 2 μL. Mass spectrometric detection was carried out with an electrospray ionization (ESI) source operated in positive ion mode under the following optimized parameters: ion source voltage 5.5 kV, ion source temperature 550 °C, curtain gas 241.3 kPa (35 psi), nebulizer gas and auxiliary gas pressures 344.7 kPa (50 psi) or swainsonine quantification; the selected reaction monitoring (SRM) transitions were quantitative ion pair (*m*/*z*) 174.3/156.0 (collision energy 19 V), qualitative ion pair (*m*/*z*) 174.3/138.2 (collision energy 26 V), and declustering potential 60 V.

### 2.7. Poisoning Hotspot Compilation and Environmental Factor Analysis

Locations of locoweed poisoning incidents of herbivores were compiled from the China National Knowledge Infrastructure (CNKI) and Web of Science (Supplementary Table S5). Climatic variables for each site were extracted from WorldClim (v2.1) at 2.5 min resolution, including the following: bio1: annual mean temperature (°C); bio2: mean diurnal temperature range (°C); bio3: isothermality; bio4: temperature seasonality; bio5: maximum temperature of warmest month (°C); bio6: minimum temperature of coldest month (°C); bio7: Temperature annual range (°C); bio8: mean temperature of wettest quarter (°C); bio9: mean temperature of driest quarter (°C); bio10: mean temperature of warmest quarter (°C); bio11: mean temperature of coldest quarter (°C); bio12: annual precipitation (mm); bio13: precipitation of wettest month (mm); bio14: precipitation of driest month (mm); bio15: precipitation seasonality (mm); bio16: precipitation of wettest quarter (mm); bio17: precipitation of driest quarter (mm); bio18: precipitation of warmest quarter (mm); bio19: precipitation of coldest quarter (mm); alt: altitude; srad: solar radiation (kJ m^−2^ day^−1^).

Principal component analysis (PCA) and Euclidean distance calculations were performed in R (v4.3.1) using the *vegan* and *factoextra* packages, with results visualized via *ggplot2* package.

### 2.8. Statistical Analysis

Statistical analysis of experimental data was performed through SPSS v 17.0; the figures were created using Graphpad (v10.2).

## 3. Results

### 3.1. Endophytic Fungus Carriage Rate and Germination Rate of Seeds

**Figure 1:**
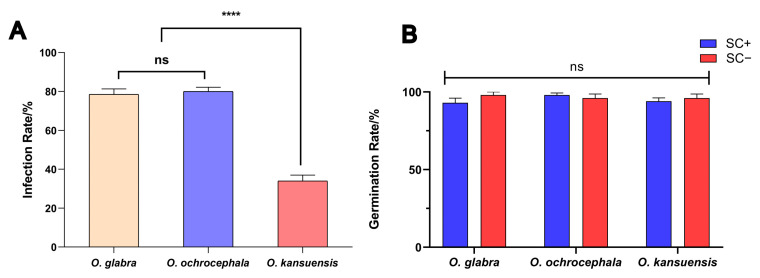
Endophytic fungus carriage rate, germination rate of seedling. (**A**) Fungal endophyte carrier rate of seeds of *O. glabra*, *O. kansuensis*, and *O. ochrocephala*. (**B**) Germination of SC+ and SC− seeds of *O. glabra*, *O. ochrocephala*, and *O. kansuensis*. Note: ns indicates insignificant difference in Duncan test (*p* > 0.05). **** indicates significant difference in Duncan test (*p* < 0.0001).

The endophytes were found on SC+ seeds (seeds with seed coats) (Figure S1). In SC+ seeds, the endophyte infection rate was not significantly (*p* ˃ 0.05) different between *O. glabra* seeds (78.5%) and *O. ochrocephala* seeds (80.0%) (Figure 1A). The endophyte infection rates of *O. kansuensis* were significantly lower than the rates of *O. glabra* seeds and *O. ochrocephala* (*p* < 0.0001). All SC− seeds of three *Oxytropis* species had no endophytic fungi.

The germination and survival rates of SC+ and SC− seedlings were measured and compared (Figure S2). The seed germination rates and the seedling survival rates were not significantly different between the SC+ and SC− groups (*p* ˃ 0.05). For instance, the germination rates of *O. glabra*, *O. ochrocephala*, and *O. kansuensis* were 70.0–100.0%, 80.0–100.0%, and 80.0–100.0%, respectively (Figure 1B).

### 3.2. Endophytic Fungus Carrier Rate and Distribution in Plants

**Figure 2:**
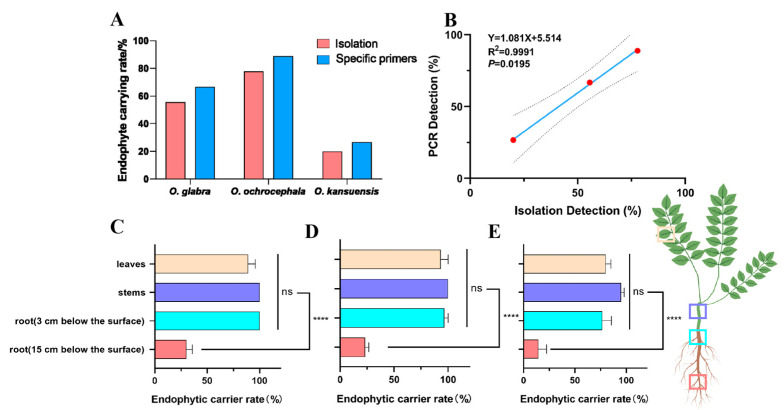
E+ and E− plants re-confirming and endophyte distribution in E+ plants. (**A**) Endophyte carrier rate of SC+ plant by isolation and specific primers of *O. glabra*, *O. ochrocephala*, and *O. kansuensis*. (**B**) Correlation analysis of isolation and PCR detection of SC+ plants. (**C**) Endophyte carrier rate in different issues of *O. glabra*. (**D**) Endophyte carrier rate in different issues of *O. ochrocephala*. (**E**) Endophyte carrier rate in different issues of *O. kansuensis*. Note: ns indicates insignificant difference in Duncan test (*p* > 0.05). **** indicates significant difference in Duncan test (*p* < 0.0001).

The isolation and PCR amplification results showed that all three species germinating from SC− seeds after four months of cultivation did not have endophytic fungi (Figures S3 and S4). And the isolation results showed that SC+ plants of *O. glabra*, *O. ochrocephala*, and *O. kansuensis* contained 55.6%, 77.8%, and 20.0% of endophytic fungi (Figure 2A and Figure S5). The PCR amplification results showed that SC+ plants of *O. glabra*, *O. ochrocephala*, and *O. kansuensis* contained 66.7%, 88.9%, and 26.7% of endophytic fungi, respectively (Figure 2A and Figure S6). The isolation rate and PCR amplification revealed a significant linear correlation (*p* = 0.0195) (Figure 2B).

The endophytic carrier rates were significantly lower in the distal root segments (14.3–30%) compared to the root collar (76.6–100%), stems (95–100%), and leaves (85–93.3%) across all three *Oxytropis* species (*p* < 0.05) (Figure 2C–E). Notably, the stem tissues showed the highest endophyte infection frequency.

### 3.3. Effect of Endophytic Fungi on Growth of E+ Plants and E− Plants

**Figure 3:**
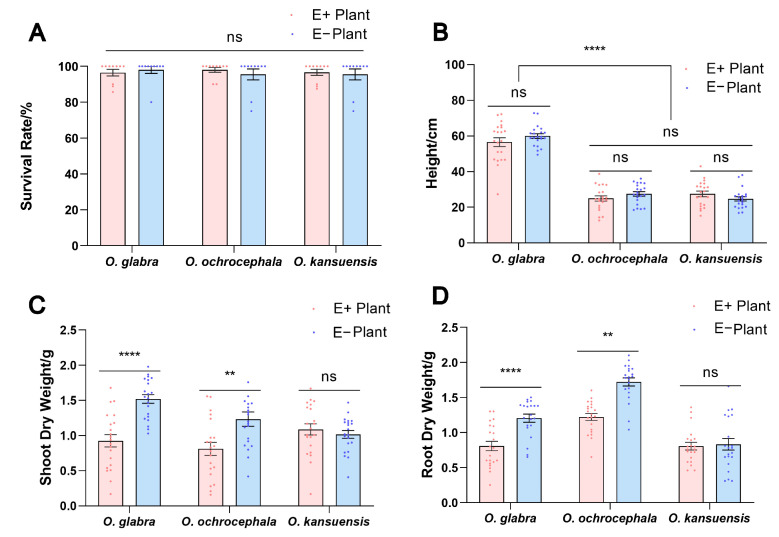
Effect of endophytic fungi on growth of E+ plants and E− plants. (**A**) Survival rate of E+ and E− plants of *O. glabra*, *O. ochrocephala*, and *O. kansuensis*. (**B**) Height of E+ and E− plants of *O. glabra*, *O. ochrocephala*, and *O. kansuensis*. (**C**) Shoot dry weight of E+ and E− plants of *O. glabra*, *O. ochrocephala*, and *O. kansuensis*. (**D**) Root dry weight of E+ and E− plants of *O. glabra*, *O. ochrocephala*, and *O. kansuensis*. Note: ns indicates insignificant difference in Duncan test (*p* > 0.05). ** indicates significant difference in Duncan test (*p* < 0.01), **** means *p* < 0.0001.

The average survival rates for E+ and E− seedlings were 96.5% and 98.0% for *O. glabra*; 98.0% and 95.5% for *O. ochrocephala*; and 96.6% and 95.5% for *O. kansuensis* (Figure 3A). Based on the detection results of *A. oxytropis* in *Oxytropis* plants, the plants carrying endophytic fungi were designated as E+ plants, while those without detected endophytic fungi were designated as E− plants for subsequent experiments.

The plant heights were not significantly different between E+ and E− plants of each species (*p* ˃ 0.05) (Figure 3B). The height of *O. glabra* was higher than other species (*p* < 0.0001). The average plant heights of E+ and E− were 56.6 cm and 60.0 cm for *O. glabra;* 25.0 cm and 27.5 cm for *O. ochrocephala;* and 27.5 cm and 24.7 cm for *O. kansuensis*.

The shoot dry weights were significantly different between E+ and E− plants for *O. glabra* and *O. ochrocephala* (*p* < 0.01) (Figure 3C). However, the plant shoot dry weights were not significantly different between E+ and E− plants for *O. kansuensis* (*p* > 0.05). The average plant shoot dry weights of E+ and E− were 0.93 g and 1.52 g for *O. glabra*, 0.81 g and 1.23 g for *O. ochrocephala,* and 1.09 g and 1.02 g for *O. kansuensis*.

The root dry weights of E− plants were significantly higher than that of E+ plants for *O. glabra and O. ochrocephala* (*p* < 0.05) (Figure 3D). However, the root dry weights were not significantly different between E+ and E− plants of *O. kansuensis* (*p* ˃ 0.05). The average plant root dry weights for E+ and E− were 0.93 g and 1.52 g for *O. glabra*; 0.81 g and 1.23 g for *O. ochrocephala*; and 1.09 g and 1.02 g for *O. kansuensis*.

### 3.4. Effect of Endophytic Fungi on Photosynthetic Parameters of E+ Plants and E− Plants

**Figure 4:**
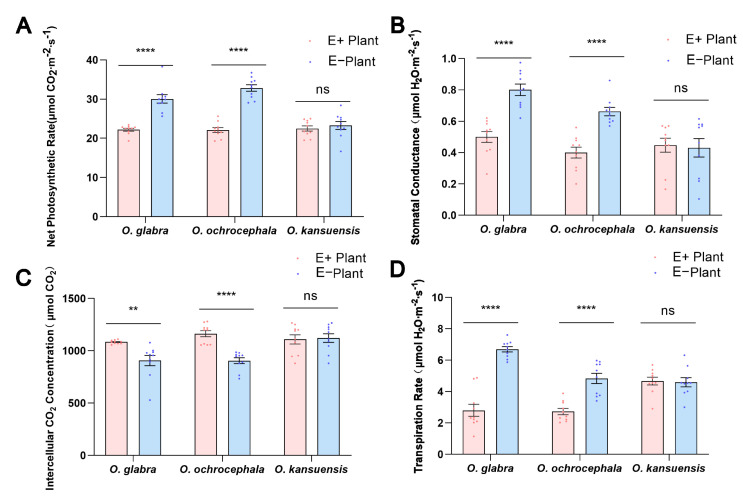
Effect of endophytic fungi on photosynthetic parameters of E+ plants and E− plants. (**A**) Net photosynthetic rate of E+ and E− plants of *O. glabra*, *O. ochrocephala*, and *O. kansuensis*. (**B**) Stomatal conductance (Gs) of E+ and E− plants of *O. glabra*, *O. ochrocephala* and *O. kansuensis*. (**C**) Intercellular CO_2_ concentration (Ci) of E+ and E− plants of *O. glabra*, *O. ochrocephala*, and *O. kansuensis*. (**D**) Transpiration rate (Tr) of E+ and E− plants of *O. glabra*, *O. ochrocephala*, and *O. kansuensis*. Note: ns indicates insignificant difference in Duncan test (*p* > 0.05). ** indicates significant difference in Duncan test (*p* < 0.01), **** means *p* < 0.0001.

The net photosyntheticrate (Pn) of E− plants after four months of cultivation in the greenhouse was significantly higher than that of E+ plants for *O. glabra* and *O. ochrocephala* (*p* < 0.0001) (Figure 4A). The average Pn of E+ and E− plants were 22.2 and 30.0 μmol CO_2_∙m^−2^∙s^−1^ for *O. glabra*, 22.1 and 32.8 μmol CO_2_∙m^−2^∙s^−1^ for *O. ochrocephala*, and 22.5 and 23.2 μmol CO_2_∙m^−2^∙s^−1^ for *O. kansuensis* (*p* > 0.05).

The stomatal conductance (Gs) of E− plants was significantly higher than that of E+ plants for *O. glabra* and *O. ochrocephala* (*p* < 0.0001) (Figure 4B). For instance, the average Gs in E+ and E− plants were 0.50 and 0.80 mol H_2_O∙m^−2^∙s^−1^ for *O. glabra*, 0.40 and 0.66 μmol H_2_O∙m^−2^∙s^−1^ for *O. ochrocephala,* and 0.45 and 0.43 mol H_2_O∙m^−2^∙s^−1^ for *O. kansuensis*.

The intercellular CO_2_ concentration (Ci) of E+ plants was significantly higher than that of E− plants for *O. glabra* and *O. ochrocephala* (*p* < 0.01) (Figure 4C). The average Ci in E+ and E− plants was 1084.3 and 907.7 μmol CO_2_∙mol^−1^ for *O. glabra*, 1163.4 and 906.1 μmol CO_2_∙mol^−1^ for *O. ochrocephala*, and 1109.3 and 1090.0 μmol CO_2_∙mol^−1^ for *O. kansuensis* (*p* > 0.05).

The transpiration rate (Tr) was significantly different between E+ and E− plants of *O. glabra* and *O. ochrocephala* (*p* < 0.05) (Figure 4D). The average Tr in E+ and E− plants was 2.8 and 6.7 μmol H_2_O∙m^−2^∙s^−1^ for *O. glabra*, 2.7 and 4.8 μmol H_2_O∙m^−2^∙s^−1^ for *O. ochrocephala*, and 4.7 and 4.6 μmol H_2_O∙m^−2^∙s^−1^ for *O. kansuensis*.

### 3.5. The Swainsonine of E+ and E− Plants

**Figure 5:**
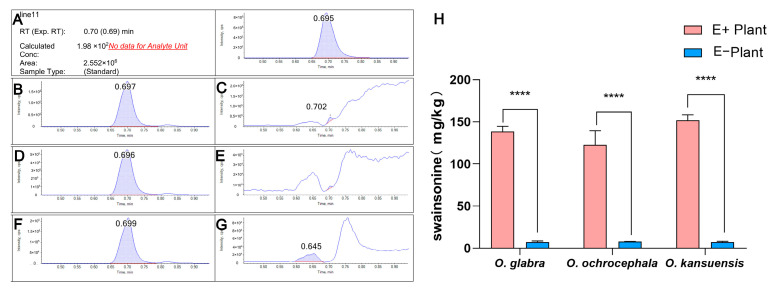
Swainsonine content of E+ and E− plants of *O. glabra*, *O. ochrocephala*, and *O. kansuensis*. (**A**) Multiple reaction monitoring (MRM) chromatogram of swainsonine standard. (**B**) MRM chromatogram of *O. glabra* E+ plant extract. (**C**) MRM chromatogram of *O. glabra* E+ plant extract. (**D**) MRM chromatogram of *O. ochrocephala* E+ plant extract. (**E**) MRM chromatogram of *O. ochrocephala* E− plant extract. (**F**) MRM chromatogram of *O. kansuensis* E+ plant extract. (**G**) MRM chromatogram of *O. kansuensis* E− plant extract. (**H**) Swainsonine content of E+ and E− plants of *O. glabra*, *O. ochrocephala*, and *O. kansuensis*. Note: **** indicates significant difference in Duncan test (*p* < 0.0001).

In three species of *Oxytropis* species, swainsonine was consistently detected in E+ plants, while being rarely or undetectable in E− plants (Figure 5A–G). The average swainsonine of E+ plants was 122.6–151.7 mg/kg (Figure 5H). The average swainsonine of E− plants was 7.3–7.8 mg/kg, which was extremely low. The swainsonine of E+ plants was remarkably higher than E− plant (*p* < 0.0001).

### 3.6. Association of Environmental Factors with Livestock Poisoning Incidents Induced by Locoweed

**Figure 6:**
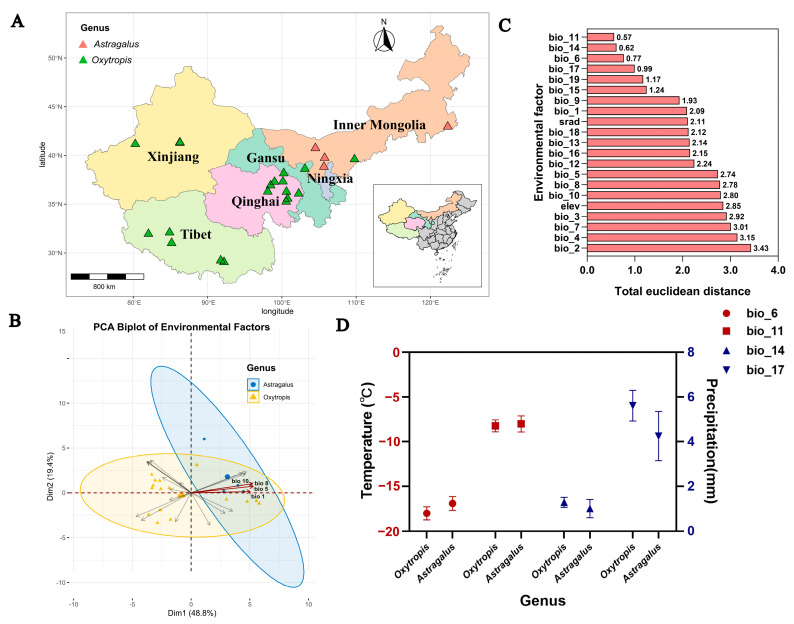
Environmental factors associated with livestock poisoning hotspots caused by locoweed. (**A**) Geographic distribution of livestock poisoning hotspots. (**B**) PCA biplot of environmental factors at livestock poisoning hotspots. (**C**) Euclidean distances of environmental factors among livestock poisoning hotspots. (**D**) Four common environmental characteristics (mean ± SEM) shared by locoweed.

According to literature records, 22 severe livestock poisoning incidents caused by *Oxytropis* and *Astragalus* have been documented in Xinjiang, Qinghai, Tibet, Gansu, and Inner Mongolia (Table S1, Figure 6A). A principal component analysis (PCA) biplot of environmental factors at the poisoning sites revealed no distinct population stratification among the two genera (Figure 6B). Specifically, *Oxytropis* populations were distributed across all four quadrants, while Astragalus primarily clustered in Quadrant I. The PCA biplot showed Dimension 1 (Dim.1; explaining 48.8% variance) was predominantly associated with temperature-related variables showing high contributions (COS^2^ > 0.8): maximum temperature of warmest month (Bio5, COS^2^ = 0.95), mean temperature of wettest quarter (Bio8, COS^2^ = 0.93), mean temperature of warmest quarter (Bio10, COS^2^ = 0.93), and annual mean temperature (Bio1, COS^2^ = 0.86). Dimension 2 (Dim.2; 19.4% variance explained) exhibited limited variable contributions (COS^2^ = 0.0006–0.47), indicating substantial noise and limited interpretability, and was thus excluded from further analysis (Figure 6B). This spatial pattern suggests that *Astragalus* thrive under warmer seasonal conditions.

To identify shared environmental features across poisoning sites, we calculated inter-hotspot Euclidean distances for each environmental variable (Figure 6C). The smallest inter-hotspot distances (Euclidean distance < 1) were observed for mean temperature of coldest quarter (Bio11, 0.57), precipitation of driest month (Bio14, 0.62), minimum temperature of coldest month (Bio6, 0.77), and precipitation of driest quarter (Bio17, 0.99). Common environmental characteristics (mean) across two genera included −17.31 °C of minimum temperature of coldest month, −7.91 °C of mean temperature of coldest quarter, 1.09 mm of precipitation of driest month, and 4.95 mm of precipitation of driest quarter (Figure 6D).

Abbreviations: bio1: annual mean temperature (°C); bio2: mean diurnal temperature range (°C); bio3: isothermality; bio4: temperature seasonality; bio5: maximum temperature of warmest month (°C); bio6: minimum temperature of coldest month (°C); bio7: temperature annual range (°C); bio8: mean temperature of wettest quarter (°C); bio9: mean temperature of driest quarter (°C); bio10: mean temperature of warmest quarter (°C); bio11: mean temperature of coldest quarter (°C); bio12: annual precipitation (mm); bio13: precipitation of wettest month (mm); bio14: precipitation of driest month (mm); bio15: precipitation seasonality (mm); bio16: precipitation of wettest quarter (mm); bio17: precipitation of driest quarter (mm); bio18: precipitation of warmest quarter (mm); bio19: precipitation of coldest quarter (mm); alt: altitude; srad: solar radiation (kJ m^−2^ day^−1^).

## 4. Discussion

### 4.1. Transmission of Endophyte Alternaria Oxytropis in Different Hosts

*Alternaria oxytropis* is classified as a Class 1 endophyte, characterized by its narrow host specificity to locoweeds and predominant vertical transmission [32]. Our study confirms the dominance of maternal transmission in *A. oxytropis*. Seed coat removal (SC− treatment) effectively eliminated endophytes without compromising seed viability (Supplementary Figures S1 and S2), aligning with prior reports of seed coat-localized transmission in locoweeds [11,33].

Notably, the endophyte carriage rate declined from SC+ seeds to mature plants in *O. glabra* and *O. kansuensis*, mirroring Cook et al.’s [34] observation of higher seed infection rates (80–100%) than resultant plants (14%). This suggests incomplete vertical transmission efficiency. Intriguingly, *O. ochrocephala* displayed the opposite trend, with higher plant infection rates (88.9%) than seeds (80.0%), potentially indicating cryptic horizontal transmission or post-germination colonization—a phenomenon warranting further investigation, as Braun et al. [11] similarly reported elevated stem infection rates (97.2%).

Endophyte prevalence significantly differed among species, with *O. kansuensis* showing markedly lower carriage rates than *O. glabra* and *O. ochrocephala* (Figure 2A). This disparity may reflect host genotype–endophyte compatibility or environmental modulation [35], though the mechanistic basis requires elucidation.

### 4.2. The Influence of Endophye Alternaria Oxytropis on Plant Growth

Endophytic fungi typically form mutualistic symbioses with host plants, wherein the fungi obtain nutrients and habitat from the host while conferring benefits such as enhanced resistance to herbivores, pathogens, and abiotic stresses [34,36,37,38,39,40]. However, *Alternaria* section *Undifilum* has long been considered an exception, as it appears to provide no clear growth benefits to locoweed hosts. For instance, Klypina et al. [27] found that endophytes reduced shoot and root biomass of American locoweeds (*A. mollissimus* and *O. sericea*). Nevertheless, the physiological effects of *A. oxytropis* on major Chinese locoweed species remained unexplored prior to this study.

In this study, the effects of *A. oxytropis* on *O. glabra*, *O. ochrocephala*, and *O. kansuensis* plants were evaluated under greenhouse conditions. It was worth noting that endophytic fungi exhibited a negligible impact on the growth indices of *O. kansuensis*, suggesting their limited influence on its growth dynamics. This observation could potentially account for the relatively low prevalence of endophytic fungi within the population of *O. kansuensis*. Further, *A. oxytropis* infection significantly reduced photosynthetic parameters (net photosynthesis rate, stomatal conductance, and transpiration rate) while increasing intercellular CO_2_, indicating impaired photosynthetic efficiency and likely contributing to the observed biomass reduction.

The tissue distribution of *A. oxytropis* within the host aligns with these findings. The endophyte preferentially colonized aboveground tissues (85–100% infection frequency) over roots (14.3–30%) (Figure 2C–E), which is consistent with reports of seasonal swainsonine accumulation in photosynthetic organs of *O. sericea* [41]. During vegetative growth (as in our study), endophytes and swainsonine primarily localize to leaves and stems. Notably, E+ plants harbored 16–20 times higher swainsonine concentrations than E− plants (Figure 5H), likely as an anti-herbivore adaptation—though field grazing trials are needed for validation [9]. Based on these results, we hypothesize that reduced photosynthesis in E+ plants limits photoassimilate availability, diverting resources toward fungal growth and swainsonine synthesis at the expense of host biomass.

The mechanism by which *A. oxytropis* reduces photosynthesis in locoweed hosts remains unclear. Only a few studies reported that endophytic fungi reduced photosynthesis. For instance, Wężowicz et al. found that endophyte *Diaporthe* spp. decreased the photosynthesis of *Verbascum lychnitis* due to a significant decline in the efficiency of primary photochemistry (φEo, φPo), suggesting that solar energy was not transformed into fixed energy but instead was dissipated as heat (DIo/RC) [42]. However, further studies are needed to examine whether the mechanism of *A. oxytropis* in this study is related to the photosynthesis reduction [42].

Interestingly, although endophytes reduced shoot biomass, they had no effect on the height of the host plant, indicating that endophytes did not affect the elongation of the stem. Guan et al. [25] found that *A. oxytropis* increased the concentrations of plant endogenous auxin and the expression of key genes for auxin biosynthesis, signaling, and transport. Thus, the preservation of stem growth in plants may reflect endophyte-mediated auxin promotion.

### 4.3. Poisoning Hotspots of Herbivores Reveal Enhanced Symbiont Performance by A. oxytropis Under Extreme Winter Stress

Though *A. oxytropis* has negative and neutral effects on the growth of predominant locoweeds, the endophyte–locoweed symbionts are widely distributed (nearly 100% of endophytic fungi carrying rate) in China and the USA, which indicates that the symbionts have strong adaptability. This apparent paradox can be explained by context-dependent benefits: while the endophyte reduces host biomass under well-watered conditions, it confers critical advantages in stressful environments, e.g., water deficit stress [27]. We propose that under stress, symbiotic benefits exceed maintenance costs (e.g., fungal growth and swainsonine biosynthesis), ultimately enhancing symbiont fitness [42]. Field evidence supports this—*A. oxytropis* symbiosis predominates in arid regions but is occasionally lost in high-precipitation areas [43].

The clustering of livestock poisoning hotspots largely reflects both the high prevalence of *A. oxytropis* in these hosts and its competitive advantage over other plants, indicating strong environmental selection favoring the symbiont. These regions share extreme dry and cold winter conditions: minimum temperatures of −17.31 °C (coldest month) and −7.91 °C (coldest quarter), with minimal precipitation (1.09 mm and 4.95 mm in the driest month and quarter, respectively).

While most legumes survive winter through root dormancy after aerial senescence, such conditions risk cellular dehydration and ice damage [44]. Concurrently, winter drought reduces shallow and deep soil water availability in subsequent seasons, adversely affecting the water balance of entire plant communities [45]. Notably, *Alternaria* section *Undifilum* colonized shoots during the growing season [41], while persisting in roots over winter, ensuring recurrent host infection [46]. Klypina et al. [27] found that under sufficient water, *A. cinereum* reduced the dry weight of *Astragalus mollissimus*, while *A. cinereum* had no effect on the dry weight of plants under drought stress. *A. oxytropis* enhances drought tolerance in hosts by promoting lateral root and root hair development, through upregulating endogenous auxin levels and modulating the expression of auxin biosynthesis, signaling, and transport genes [25]. This mechanism may explain the symbiont’s success in harsh environments. Furthermore, drought stress amplifies toxicity in *Alternaria*-colonized *A. mollissimus* [27], potentially explaining the association between livestock poisoning hotspots and arid environments.

While endophytes like *Ewingella* sp. have been shown to enhance cold tolerance in *Colobanthus quitensis* through polyphenol metabolism reprogramming and accumulation of stress-related metabolites [47], the cold–drought adaptation mechanisms mediated by *Alternaria* sect. *Undifilum* in locoweeds remain poorly understood, warranting further investigation into symbiotic performance under combined winter stress.

### 4.4. Limitations and Future Directions

While this study investigated endophyte effects on three main locoweed species under controlled greenhouse conditions, its applicability to natural ecosystems requires careful consideration. Locoweeds in northern China experience substantially more complex environmental variations than laboratory settings can replicate, potentially altering endophyte–host interactions in field populations. Although we confirmed exclusive colonization by *A. oxytropis* through WA medium culturing and specific primer detection, the potential influence of unculturable endophytes on our results cannot be excluded. Although poisoning hotspot analyses suggested that endophytes benefit symbionts under extreme winter stress (−17.3 °C to −7.9 °C, <5 mm precipitation), these ecological patterns need verification through further greenhouse and field experiments. Moreover, the observed host-specific responses—from significant growth inhibition in *O. glabra*/*O. ochrocephala* to neutral effects in *O. kansuensis*—point to uncharacterized molecular mechanisms governing these species-dependent symbioses that warrant further investigation.

## 5. Conclusions

In this study, we compared growth and photosynthesis in endophyte-infected and -free plants of three dominant Chinese locoweed species (*Oxytropis kansuensis*, *O. glabra*, and *O. ochrocephala*) and analyzed environmental factors at herbivore poisoning hotspots to investigate the impact of *Alternaria oxytropis*. Under controlled conditions, the endophyte significantly reduced photosynthetic efficiency and biomass (26.0–38.8% in *O. glabra*; 32.6–34.1% in *O. ochrocephala*) while increasing intercellular CO_2_, suggesting a metabolic cost associated with swainsonine production (122.6–151.7 mg/kg in E+ plants). In contrast, *O. kansuensis* exhibited neutral growth responses, potentially linked to its lower endophyte colonization rate (20.0–26.7% vs. 55.6–88.9% in other species), highlighting host-specific compatibility in this symbiosis.

The paradox of widespread endophyte prevalence, despite its growth-limiting effects, may be resolved by environmental context. Poisoning hotspot analyses revealed that symbiont dominance correlates with extreme winter conditions (minimum temperatures ≤ −17 °C and precipitation ≤ 1 mm during the driest month), suggesting that *A. oxytropis* enhances locoweed fitness under abiotic stress. While these findings suggest that the effects of endophytes on hosts vary with host species and environment, the observed correlations require verification through controlled stress experiments and field validation to establish causal relationships and elucidate the underlying physiological mechanisms.

## Data Availability

The original contributions presented in this study are included in the article/Supplementary Materials. Further inquiries can be directed to the corresponding author(s).

## Supplementary Materials

The following supporting information can be downloaded at: https://www.mdpi.com/article/10.3390/jof11070516/s1. Figure S1: The culture of the seeds with and without seed coats; Figure S2: Growth conditions of three species of locoweed; Figure S3: No endophytic fungi were isolated from stems cultured from peeled seeds; Figure S4: Specificity of PCR with primers OmtssuF/OmtssuR using DNA of locoweed plants grown from seeds without coats; Figure S5: Endophytic fungi grew from the section cultured from unpeeled seeds 3 days after isolation; Figure S6: Specificity of PCR with primers OmtssuF/OmtssuR using DNA of *O. ochrocephala*, *O. glabra*, and *O. kansuensis* plants grown from seeds with coats.; Table S1: Information of poisoning hotspots caused by *Oxytropis* and *Astragalus*. References [48,49,50,51,52,53,54,55,56] are cited in the Supplementary Materials.
