# Supplementary material for: Host-Specific and Environment-Dependent Effects of Endophyte Alternaria oxytropis on Three Locoweed Oxytropis Species in China
Source: J Fungi (Basel). 2025 Jul 9;11(7):516. doi: 10.3390/jof11070516 (PMC12296004; doi:10.3390/jof11070516)
Supplement: Supplementary file 1 [file jof-11-00516-s001.zip › jof-3723457-supplementary.pdf]

Supporting information

Host-Specific and Environment-Dependent Effects of Endophyte *Alternaria oxytropis* on Three Locoweed *Oxytropis* Species in China

Yue-Yang Zhang [1], Yan-Zhong Li [1,*], Zun-Ji Shi [1,*]

Contents

1. **Fig. S1** The culture of the seeds with and without seedcoat.

2. **Fig. S2** Growth conditions of three species of locoweed.

3. **Fig. S3** No endophytic fungi were isolated from stems cultured from peeled seeds.

4. **Fig. S4** Specificity of PCR with primers OmtssuF/OmtssuR using DNA of locoweed plants grown from seeds without coats.

5. **Fig. S5** Endophytic fungi grew from the section cultured from unpeeled seeds 3 days after isolation.

6. **Fig. S6** Specificity of PCR with primers OmtssuF/OmtssuR using DNA of *O. ochrocephala*, *O. glabra* and *O. kansuensis* plants grown from seeds with coats.

7. **Table S1** Information of poisoning hotspots caused by *Oxytropis* and *Astragalus*.

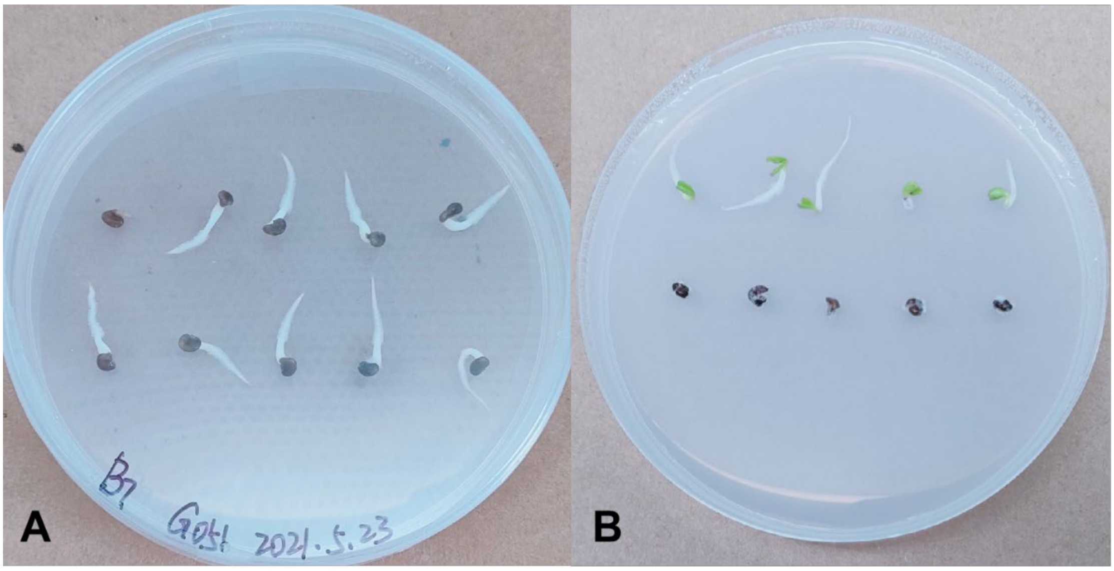

Fig. S1 The culture of the seeds with (A) and without (B) seedcoat.

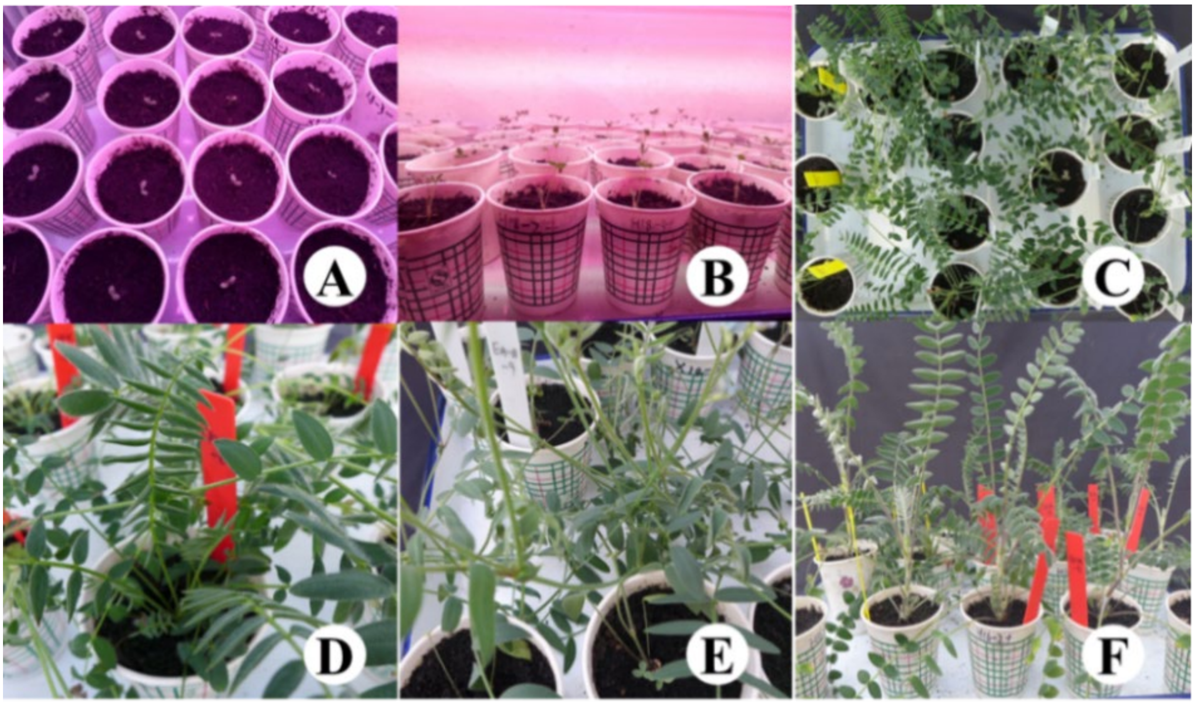

Fig. S2 Growth conditions of three species of locoweed (A: 3 days after transplantation; B: 1 week after transplantation; C and D: *O. kansuensis* and Gansu thorny pea 2 months after transplantation; E: *O. glabra* 2 months after transplantation; F: *O. ochrocephala* thorny pea 2 months after transplantation)

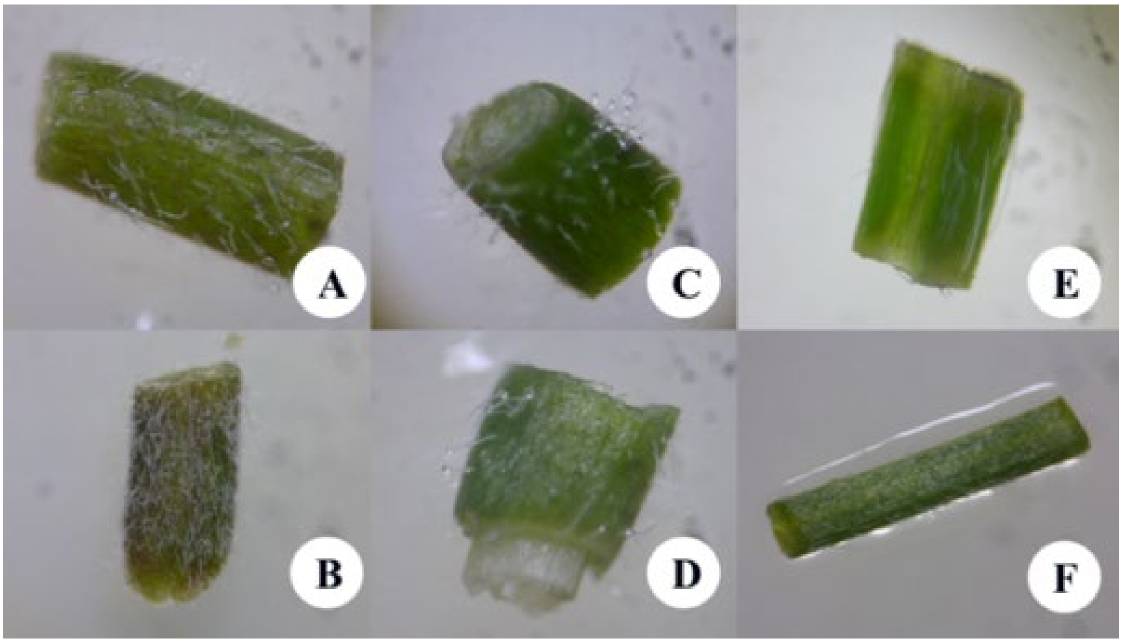

Fig. S3 No endophytic fungi were isolated from stems cultured from peeled seeds (A, B: the sample of *O. ochrocephala*; C, D: the sample of *O. kansuensis*; E, F: the sample of *O. glabra*)

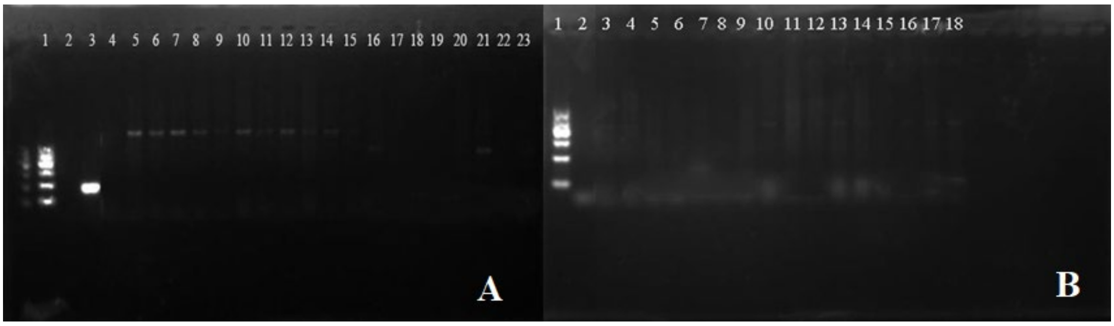

Fig. S4 Specificity of PCR with primers OmtssuF/OmtssuR using DNA of locoweed plants grown from seeds without coats. (**A** 1: DNA Marker DL1200; 2: negative control; 3: Positive control: locoweed endophytic fungi *A. oxytropis*; 4-15: H1-H12 of *O. ochrocephala* plants; 16-23: X1-X8 of *O. glabra* plants; **B** 1: DNA Marker DL1200; 2: negative control; 3-6: X9-X12 of *O. glabra* plants; 7-18: G1-G12 of *O. kansuensis* plants)

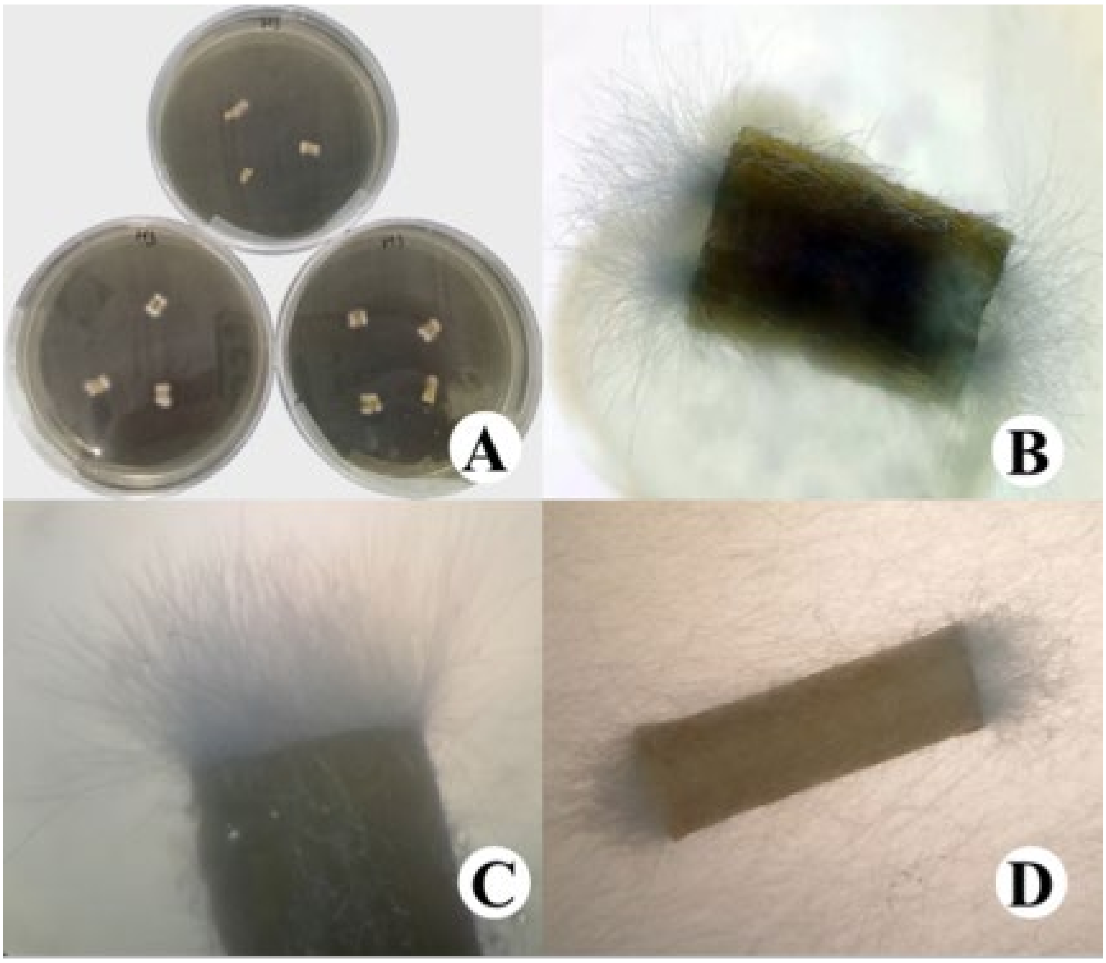

Fig. S5 Endophytic fungi grew from the section cultured from unpeeled seeds 3 days after isolation (A: Hypha grew from the section of the locoweed plant samples; B: Mycelium grew from *O. ochrocephala*; C: Hypha grew from *O. kansuensis*; D: Hypha grew from *O. glabra*)

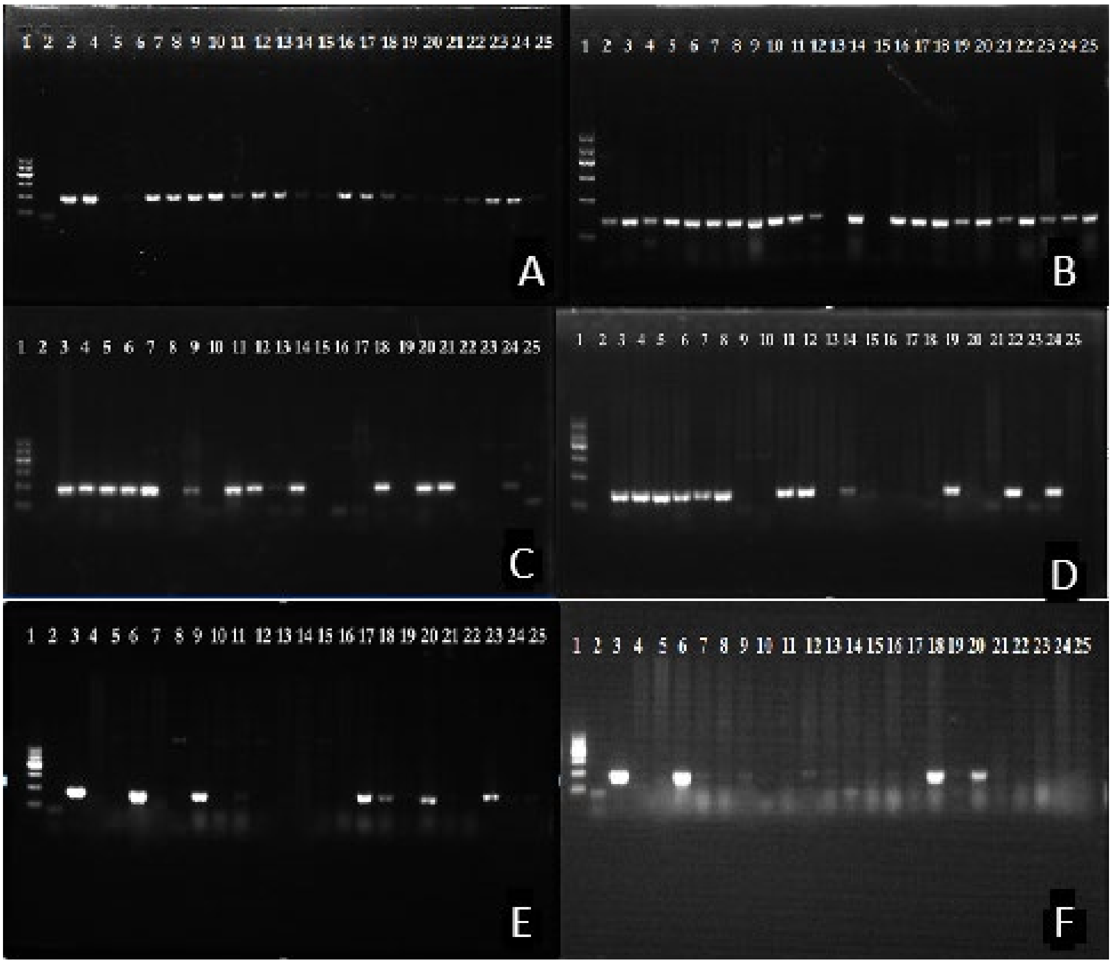

Fig. S6 Specificity of PCR with primers OmtssuF/OmtssuR using DNA of *O. ochrocephala* (A, B), *O. glabra* (C, D) and *O. kansuensis* (E, F) plants grown from seeds with coats. (**A** 1: DNA Marker DL1200; 2: negative control; 3: Positive control: endophytic fungi *A. oxytropis* isolated from *O. ochrocephala*; 4-25: *O. ochrocephala* plants; **B** 1: DNA Marker DL1200; 2-25: *O. ochrocephala* plants; **C** 1: DNA Marker DL1200; 2: negative control; 3: Positive control: endophytic fungi *A. oxytropis* isolated from *O. glabra*; 4-25: *O. glabra* plants; **D** 1: DNA Marker DL1200; 2-25: *O. glabra* plants; **E** 1: DNA Marker DL1200; 2: negative control; 3: Positive control: endophytic fungi *A. oxytropis* isolated from *O. kansuensis*; 4-25: *O. kansuensis* plants; **F** 1: DNA Marker DL1200; 2-25: *O. kansuensis* plants)

Table S1 Information of poisoning hotspots caused by *Oxytropis* and *Astragalus*

Poisoning Site	Latitude	Longitude	Genera	Reference
Hailong county, Qinghai	36.1009339	102.2651586	*Oxytropis*	[1]
Qilian county, Qinghai	38.1769669	100.251638	*Oxytropis*	[1]
Tongde county, Qinghai	35.2566608	100.5724335	*Oxytropis*	[1]
Guinan county, Qinghai	35.5699612	100.7728091	*Oxytropis*	[1]
Gonghe county, Qinghai	36.2814975	100.6158148	*Oxytropis*	[1]
Gangcha county, Qinghai	37.3267156	100.1402485	*Oxytropis*	[1]
Tianjun county, Qinghai	37.2973834	99.0137987	*Oxytropis*	[1]
Wulan county, Qinghai	36.925695	98.4779679	*Oxytropis*	[1]
Dulan county, Qinghai	36.3024735	98.0951591	*Oxytropis*	[1]
Gê'gyai county, Tebit	31.9521669	82.0239203	*Oxytropis*	[2]
Naidong county, Tebit	29.2284265	91.7599651	*Oxytropis*	[2]
Qvsong county, Tebit	29.0658354	92.2013717	*Oxytropis*	[2]
Yikezhao Meng Ordos, Inner Mongolia	39.6081457	109.7763659	*Oxytropis*	[3]
Tarim county, Xinjiang	41.2902222	86.2710536	*Oxytropis*	[4]
Aksu county, Xinjiang	41.1686701	80.2581875	*Oxytropis*	[5]
Yuli county, Xinjiang	41.3426164	86.2577315	*Oxytropis*	[6]
Minqin county, Gansu	38.626481	103.0950181	*Oxytropis*	[7]
Gaize county, Tebit	32.098918	84.8784098	*Oxytropis*	[8]
Horqin Left Wing Rear Banner, Inner Mongolia	42.9574834	122.3345885	*Astragalus*	[9]
Alxa Left Banner, Inner Mongolia	38.832365	105.6605393	*Astragalus*	[9]
Uliji Sumu, Inner Mongolia	40.743099	104.5083488	*Astragalus*	[9]
Gillantai, Inner Mongolia	39.7557109	105.7567181	*Astragalus*	[9]

Reference

1. Zhang, S.M., Gao, Q.D., Hou, D.H., Li, O., Chen, J.M., and Zhu, X.W. *OXYTROPIS KANSUENSIS* poisoning. *Chin. J. Anim. Vet. Sci.* **1981,** *12*, 1–6+73–74.
2. Yu, Y.X., Ji, S.L., and Tian, F.Y. Main toxic plants and their control in natural grassland of Tibet. *Grassl. Sci.* **1997**, *14*, 32–33+36.
3. You, Y., Ma, Q., Guo, Y., Poriferous, I., Shi, F., Wu, C., and Zhao, B. Status and control measures of toxic grass hazards in Inner Mongolia natural grassland. *Prog. Vet. Med.* **2018**, *39*, 105–110.
4. Wang, H. Several common poisonous plants in Horqin grassland of eastern Inner Mongolia. *Chin. Grassl. Pasture* **1986**, *6*, 50–52.
5. Yan, D.; Wu, C.; Zhao, B. Research progress on the distribution and control technology of toxic grass disasters in natural grassland in China. *Guizhou Nongye Kexueguizhou Agric. Sci.* **2016**, *44*, 104–109.
6. Yan, D., Zhou, Q., Lu, H., Wu, C., Zhao, B., Cao, D., Ma, F., and Liu, X. Distribution and control measures of toxic grass disasters in Xinjiang natural grassland. *Sci. Agric. Sin.* **2015**, *48*, 565–582.
7. Tan, C. Distribution, harm and control measures of main poisonous grasses in natural grassland of Gansu province. *PrataculturaI Sci.* **2006**, *12*, 98–101. https://doi.org/10.1360/aps040178.
8. Zhao, B., Liu, Z., Huo, X., Guo, X., Wang, J., Liu, Z., Sun, L., and Shi, Z. Harm and control measures of poisonous grass in western grassland of China. *Sci. Agric. Sin.* **2008**, *41,* 3094–3103. https://doi.org/10.3864/j.issn.0578-1752.2008.10.024.
9. Danone. Research history of Locoweed in Alxa left banner. *Chin. Anim. Husb. Vet. Abstr.* **2016**, *32*, 43–44.
